# RNA-Seq based transcriptome analysis in oral lichen planus

**DOI:** 10.1186/s41065-021-00202-z

**Published:** 2021-10-06

**Authors:** Haoyu Wang, Yiwen Deng, Siqi Peng, Li Yan, Hui Xu, Qingzhong Wang, Zhengyu Shen

**Affiliations:** 1 Department of Dermatology, Shanghai Ninth People’s Hospital, Shanghai Jiao Tong University School of Medicine, Shanghai, China; 2 Department of Oral Mucosal Diseases, Shanghai Ninth People’s Hospital, College of Stomatology, Shanghai Jiao Tong University School of Medicine, National Clinical Research Center for Oral Diseases, Shanghai Key Laboratory of Stomatology & Shanghai Research Institute of Stomatology, Shanghai, China; 3 Shanghai Key Laboratory of Compound Chinese Medicines, The MOE Key Laboratory for Standardization of Chinese Medicines, Institute of Chinese Materia Medica, Shanghai University of Traditional Chinese Medicine, Shanghai, China; 4 Department of Dermatology, Shanghai Ruijin Hospital, Shanghai Jiao Tong University School of Medicine, Shanghai, China

**Keywords:** Oral lichen planus, RNA sequencing, Weighted gene co-expression network analysis, Pathogenesis

## Abstract

**Objectives:**

Oral lichen planus (OLP) is a T cell-mediated autoimmune disease recognized as an oral potential malignant disorder (OPMD) with the precise mechanism unknown. This study focused on the transcriptional profiles of OLP to elucidate its potential pathogenesis.

**Methods:**

We conducted RNA sequencing on matched 6 OLP tissues and 6 normal oral mucosal tissues. Gene Ontology (GO) enrichment analysis, Kyoto Encyclopedia of Genes and Genomes (KEGG) pathway and weighted gene co-expression network analysis (WGCNA) were performed on differentially expressed genes (DEGs). We utilized qRT-PCR to validated the top dysregulated genes and hub genes in another 10 pairs of specimens.

**Results:**

A total of 153 DEGs (*p*-values< 0.05) were detected from RNA-Seq. According to GO and KEGG analysis, the dysregulated genes were mainly related to T cell related pathway and Wnt signaling. Based on the WGCNA analysis, 5 modules with high intramodular connectivity and hub genes in each module were gained.

**Conclusions:**

RNA-Seq and bioinformatic methods offered a valuable understanding of the biological pathways and key genes in the regulation of OLP. The identified DEGs and hub genes categorized into 2 groups including T cell regulation and inflammation and Wnt signaling pathway may serve as potential novel molecular targets for therapy.

**Supplementary Information:**

The online version contains supplementary material available at 10.1186/s41065-021-00202-z.

## Introduction

Oral lichen planus (OLP), a chronic T-cell mediated inflammatory disease, could affect oral sites including lips, buccal mucosa, tongue, palate, and gums with various lesions. With a cancerization rate of 1.65% in the patients newly diagnosed as OLP clinically, this disease was classified as an oral potential malignant disorder (OPMD) by the world health organization (WHO) in 2005 [1]. Accompanied by clinical features ranging from interlacing white lines, blisters and erosions to burning tingling sensations in the mouth as well as the absence of curative treatment, OLP has brought great distress to patient’s physical and mental health, among which the reticular OLP was the most common type. To date, the lack of solid in vitro cell models to study the pathogenesis of OLP posed major challenges to the treatment of OLP, both in terms of therapeutic strategies and new drug development [2].

Researches have suggested that immune, infection, mental and psychological factors and genetics played roles in the pathogenesis of OLP [3]. Recently, increasing studies on OLP have been conducted to identify the potential molecular mechanism, mostly focusing on the transcriptional analysis of OLP and several differentially expressed genes (DEGs) have been reported using microarray experiments [4, 5]. RNA-Seq, emerging as a novel approach for gene transcriptional profiling recently, has great advantages over the traditional hybridization-based techniques like microarrays. With no need for transcript-specific probes, the full sequences could be converted into several short reads easily sequenced by RNA-Seq, thus facilitating the detection of novel transcripts. Besides, RNA-Seq is more sensitive to identify the genes with low expression and the rare transcripts could be easily detected for the increased sequencing coverage depth of this technology.

Currently, bioinformatic approaches such as Gene Ontology (GO) and weighted gene co-expression network analysis (WGCNA) have been applied to detect the roles of each gene in the occurrence and progress of the disease at the transcriptional level and have been used widely in the field of various oral diseases, including oral squamous cell carcinoma [6, 7], periodontitis [8] and oral submucous fibrosis [9]. However, to date, studies that focus on the transcriptional profiling of OLP using RNA-Seq, especially utilizing bioinformatics have been scarcely reported. As a result, further researches with expanded samples are urgently needed to find relevant genes and signaling pathways mediated by these genes, which may offer new insights into the molecular mechanism of OLP.

In this study, firstly we constructed mRNA expression profiles in OLP tissues by RNA-Seq. Subsequently, to acquire more information from the RNA-Seq data, the GO analysis and WGCNA based on the DEGs were conducted, and hub genes and enrichment pathways were identified, providing a novel overview of the potential pathogenic genes and the therapeutic target of OLP.

## Materials and methods

### Patients and samples

Six patients who were newly diagnosed with reticular OLP from September to December 2018 in the department of Oral Mucosal Diseases, Shanghai Ninth People’s Hospital were enrolled in this research, including 3 males and 3 females aged 31–47. All the subjects were diagnosed with reticular OLP by clinical manifestations and histological examinations, and had not been treated for OLP within 3 months. Meanwhile, six volunteers who underwent orthognathic surgery were selected as normal controls, including 3 males and 3 females aged 22–44. No obvious inflammation and other pathological changes were detected by department of Pathology. Subjects with systemic diseases, including but not limited to hypertension, diabetes and tumors were excluded.

Subsequently, we further collected another 10 pairs of OLP tissues and normal oral mucosal tissues for qRT-PCR validation. All the specimens were stored at − 80 °C before use. This study was approved by Institutional Review Board of Shanghai Ninth People’s Hospital (SH9H - [2018]85), and patients’ informed consent was obtained prior to sample collection.

### Total RNA extraction

Total mRNA from oral mucosal tissues was extracted with TRIzol reagent (Invitrogen, CA, USA), and then concentration and purity were evaluated by Nanodrop 2000 (Thermo Fisher, CA, USA). A ratio of 260 nm/280 nm between 1.8 and 2.2 was set as the screening standard.

### RNA-Seq

Six OLP tissues and six normal oral mucosal tissues were used for RNA sequencing. Approximately 2 μg of total RNA was extracted from each specimen and pretreated with Epicentre Ribo-zeroTM rRNA Removal Kit. Then the RNA expression profile library was constructed in line with the manufacturer’s protocol of NEBNext R Ultratdirectional RNA Library Prep Kit(NEB, USA). The steps are as follows: First, RNA was lysed into small fragments after treated with NEBNext First Strand synthesis reaction buffer at high temperature treat, and the First Strand cDNA was synthesized using random hexmer primers and M-MULv reverse transcriptase. The second strand dsDNA was then obtained and the fragment residues were converted into blunt ends by exonuclease or polymerase. Subsequently, the 3’end of each dsDNA fragment was adenylated and connected to the NEBNext adapter with hairpin structure. After purification by AMPure XP system (Beckman Coulter, Beverly, USA), DNA fragments of 150 to 200 bp in length were obtained, which were then sequenced by HiSeq 2500 (Illumina, CA, USA).

### RNA-Seq data processing and analysis

FastQC (http://www.bioinformatics.babraham.ac.uk/projects/fastqc/) was utilized to check the sequencing quality of all the sample data trimmed with the FASTX-Toolkit. The sequencing reads against the human assembly GRCh37 were mapped by TopHat (v 2.0.9). Then we utilized HTSeq to calculate the read counts for each gene in each sample. The transfer matrix method (TMM) was used to normalize the DEGs. Subsequently, DEGs between the evaluated groups were screened using the software DESeq2. Benjamini-Hochberg method was used for multiple comparison tests and *p* < 0.05 was set as the threshold. To identify the isoform variants in the significant genes mRNA, the *rSeqDiff* package on the basis of hierarchical likelihood ratio algorithm was utilized to detect differential isoform expression from OLP RNA-seq data.

### Gene ontology and enrichment analysis

To better explore the underlying biological mechanism and pathways of the DEGs, Gene Ontology (GO) enrichment analysis and Kyoto Encyclopedia of Genes and Genomes (KEGG) pathway were performed using topGO and clusterProfiler packages in R platform respectively. *P* < 0.05 was set as the threshold.

### Weighted gene co-expression network analysis

Weighted gene co-expression network analysis was conducted to generate the gene co-expression network for the DEGs obtained from the RNA-Seq results. In terms of the WGCNA guidance, firstly, an appropriate soft-threshold β was necessary by testing several candidate powers. Then, we selected 10 as the soft-thresholding power for calculation of the adjacencies, after which the adjacencies were converted into a topological overlap matrix to acquire the corresponding dissimilarity. The function *hclust* was utilized to perform cluster analysis on the expression data in the samples and the discrete samples were deleted. The package *dynamicTreeCut* helped identify a group of genes with high topological overlap index as the same module, conduct hierarchical clustering, and establish a nested hierarchical clustering tree. Subsequently, we applied the function *TopHubInEachModule* to detect the hub gene in each module with high connectivity in the weighted co-expression network. The co-expression network of modules was visualized through *igraph* package. Branches of the cluster tree and different colors were used to represent different gene modules. Finally, the personalized expression perturbation profiles (PEEPs) algorithm which characterized the heterogeneity of gene expression patterns quantitatively was used to identify expression changes within each individual.

### Verification of the mRNA expression of the top DEGs and hub genes by qRT-PCR

To validate the results of RNA-sequence and WGCNA analysis, we selected the top five upregulated and downregulated genes respectively in the mRNA profile, as well as the 5 hub genes of each module. Total mRNA from oral mucosal tissues was extracted with TRIzol reagent (TaKaRa, Japan), and then concentration and purity was evaluated by Nanodrop 2000 (Thermo Fisher, USA). After the RNA was reversely transcribed into cDNA with PrimeScript RT kit (TaKaRa, Japan) according to the instructions, SYBR Premix Ex Taq TM kit (TaKaRa, Japan) was applied for qRT-PCR, with β-actin as the endogenous control gene. The real-time quantitative PCR amplification instrument (ABI StepOne Plus) was used to detect the SYBR Green fluorescence signal level after each amplification cycle. The primer sequences of the up-mentioned genes were synthesized by Sangon, China. The relative expression levels of the candidate genes were calculated using the 2-ΔΔCt method.

### Immunohistochemistry

Immunohistochemistry was performed on 5um paraffin sections of OLP and normal oral mucosa tissues. The sections were first dehydrated in graded ethanol, and then to block the endogenous peroxidase activity, they were incubated with hydrogen peroxide/9:1 methanol solution for 10 min. The sections were then incubated with rabbit anti-human CD3 (ab16669, Abcam, USA) (1:200) overnight at 4 °C. After being rinsed in PBS for three times, the samples were overlaid with secondary antibody conjugated to biotin. 3,3-diaminobenzidine was used for color development and hematoxylin for counterstain.

## Results

### Identification of the DEGs

**Fig. 1:**
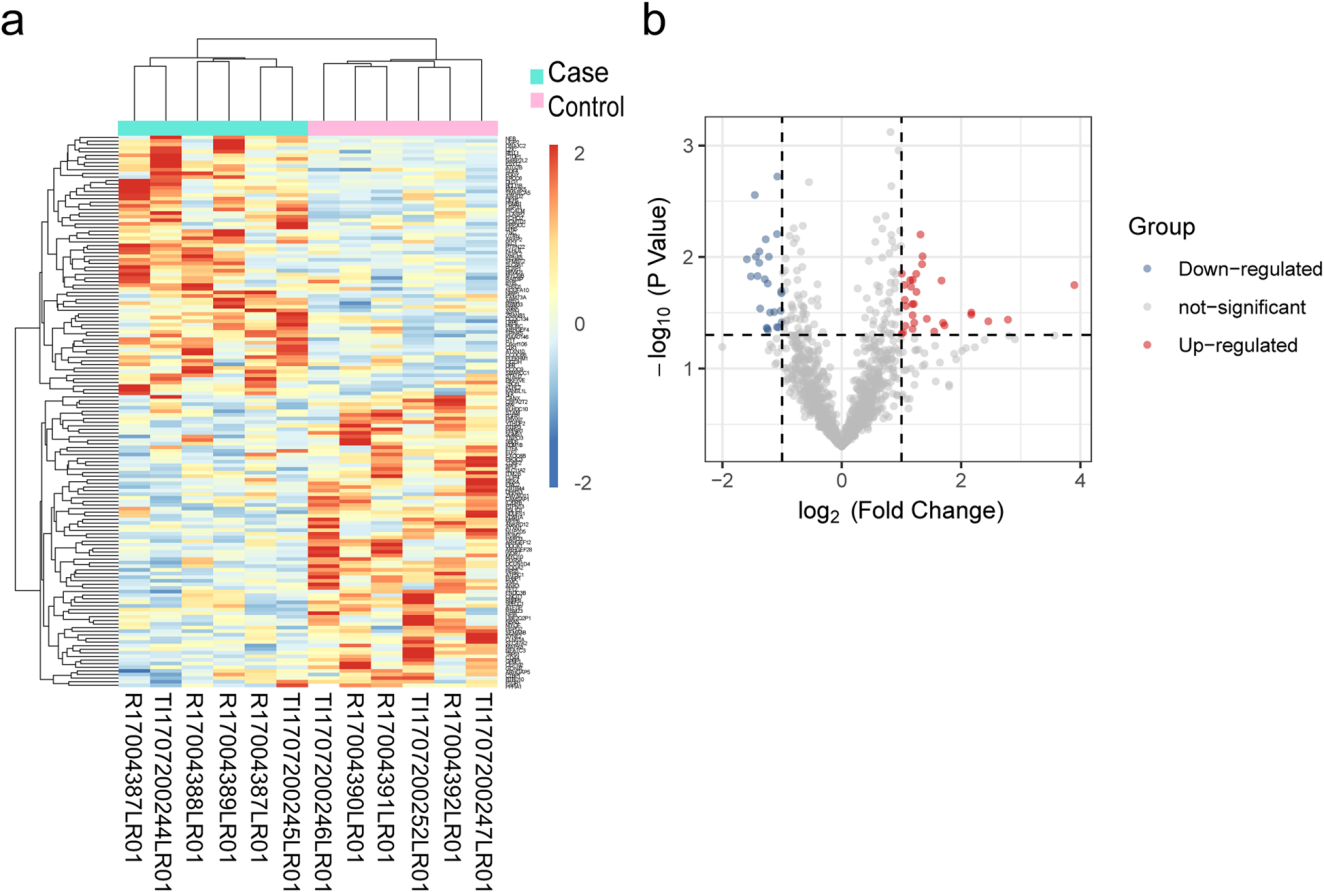
Cluster heatmap and Volcano plot of the 153 DEGs in OLP and normal oral mucosal tissues. **a** The cluster heatmap of DEGs in each sample. **b** The volcano plot of the 153 DEGs

According to high-throughput sequencing data, we obtained 153 DEGs in total with a *p*-value < 0.05 and fold change > 1.3, of which 72 were upregulated while 81 downregulated in OLP tissues against the normal oral mucosal tissues. The top 5 altered genes as well as the isoform information and statistical difference level between two comparisons have been listed in the Table S1. In addition, a heatmap and volcano plot of the 153 DEGs expression were shown in the Fig. 1. Among these genes, NEB (nebulin) was the top-ranked upregulated gene with a 14.9-fold increase.

### Gene function analysis for the dysregulated genes

**Fig. 2:**
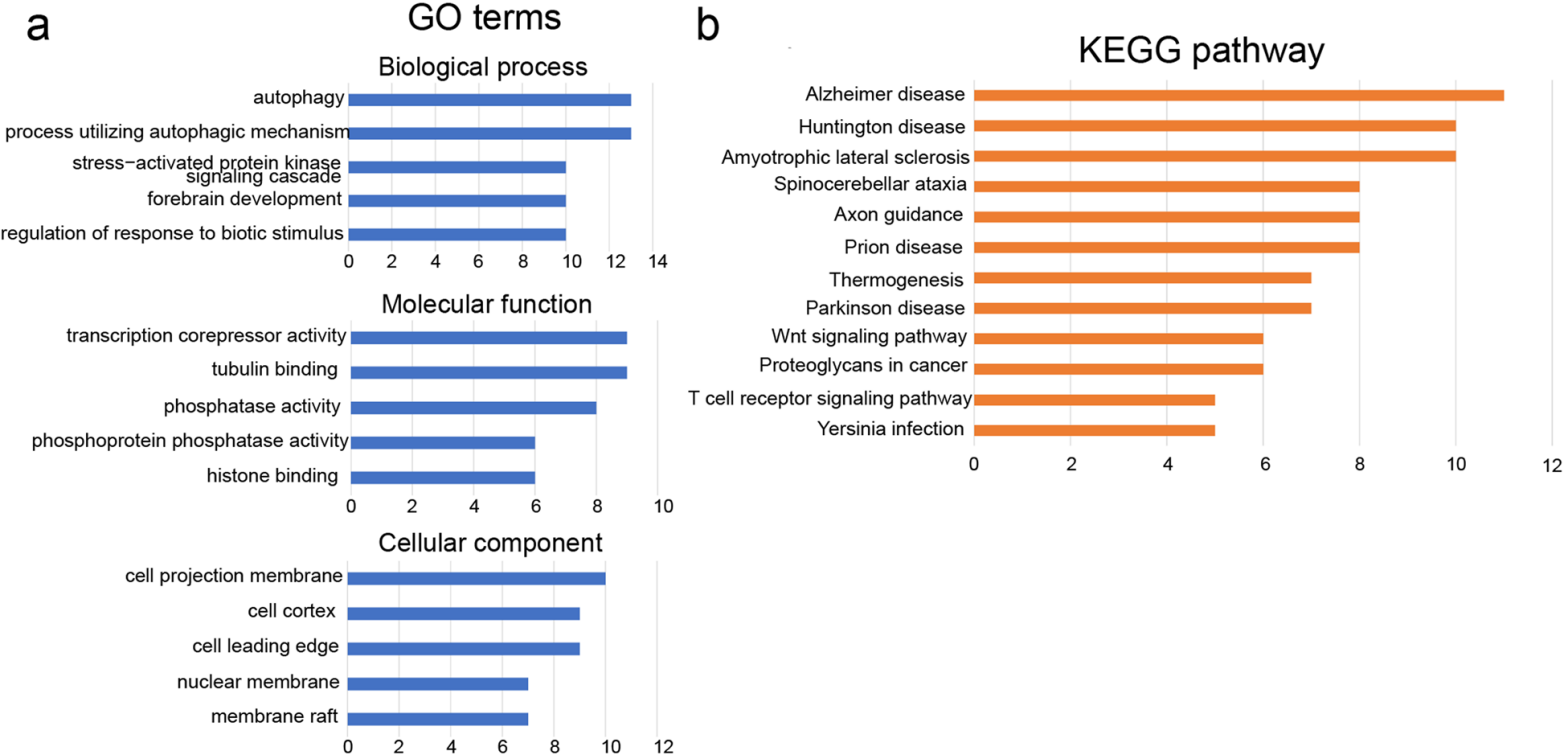
Gene Ontology enrichment analysis and KEGG pathway analysis of the DEGs. **a** The enriched GO terms of MF, CC and BP from GO analysis of the DEGs. **b** The high-enrichment KEGG pathways of the DEGs

To better explore the biological functions and pathways of the DEGs, GO and KEGG analysis were performed with topGO and clusterProfiler function. Functional annotation revealed that these DEGs were mainly related to cell regulation process. The GO analysis of the biological functions of the DEGs, including biological process (BP), cellular component (CC) and molecular function (MF) were displayed in Fig. 2a and Table S2. To be noted, these enriched terms were mainly related to autophagy process and regulation of membrane including “autophagy”, “process utilizing autophagic mechanism”, “regulation of protein localization to membrane” and “positive regulation of protein localization to membrane”.

13 KEGG pathways significantly enriched were identified in total, which were involved in T cell regulation and Wnt signaling pathway (Fig. 2b). Taken together, functional ontology analysis revealed that the significant expressed genes mainly mediated into immunoregulation process.

### Identification of hub genes in the functional modules by WGCNA

**Fig. 3:**
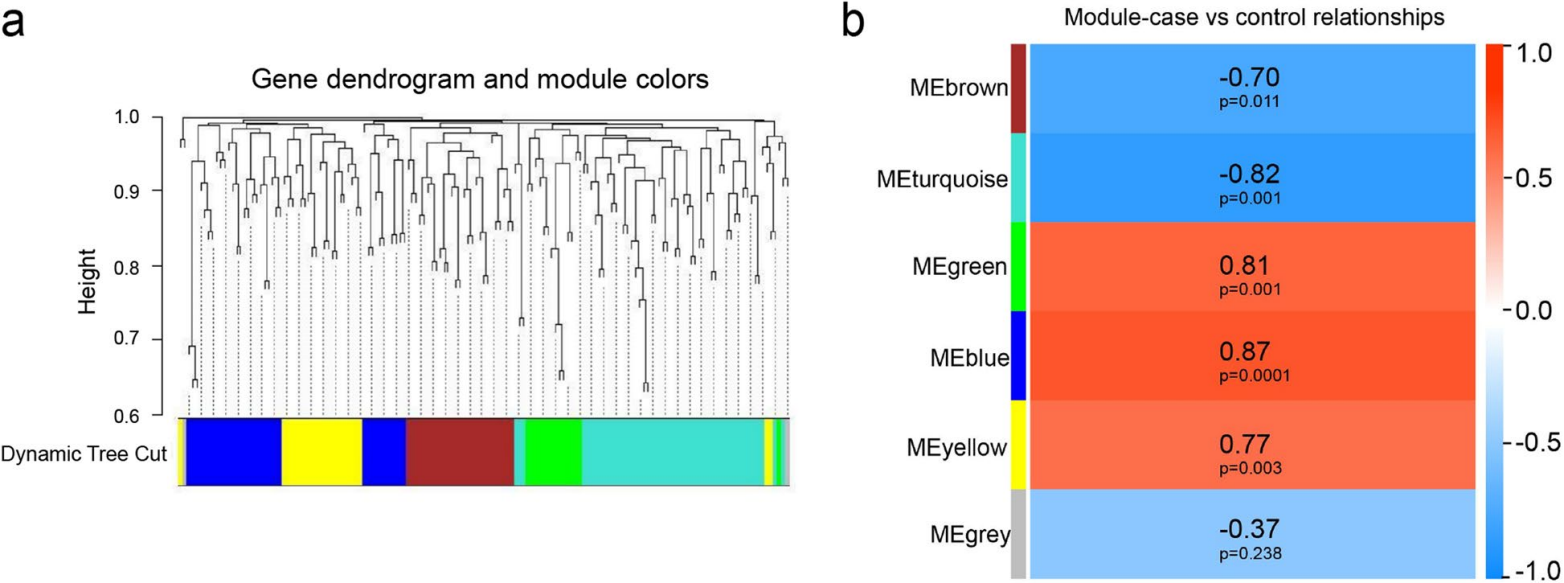
Clustering gene dendrogram plot and relationship between module and phenotypes from WGCNA. **a** The clustering gene dendrogram showing 5 co-expression modules represented by different colors in OLP. **b** Module–trait correlation analysis of the 5 co-expression modules, among which MEblue is highly correlated to OLP

**Fig. 4:**
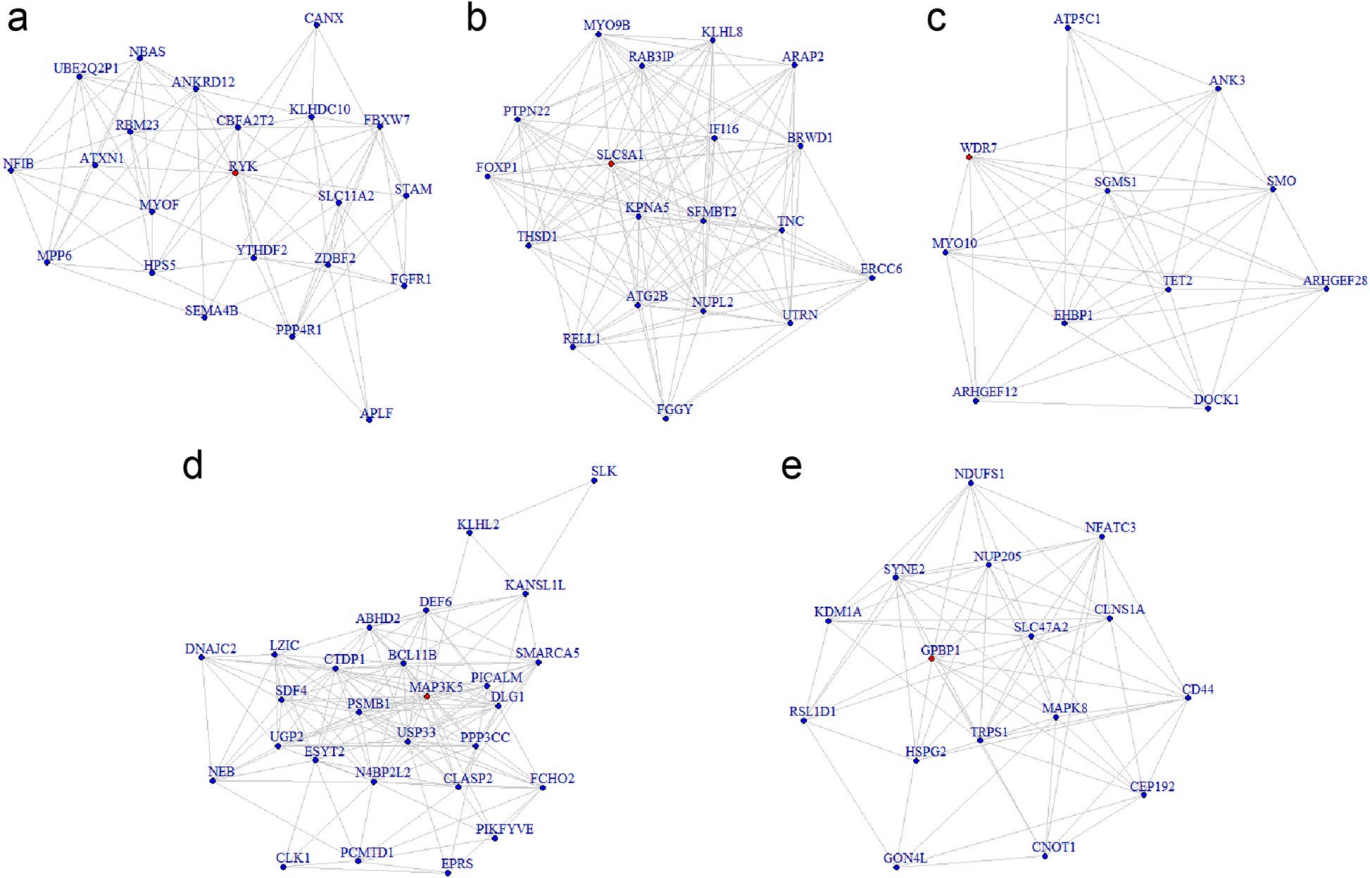
Network plots of the 5 modules with the hub genes highlighted by red nodes. **A** Network plot of the blue module with RYK as the hub gene; **B** SLC8A1 as the hub gene of the brown module; **C** WDR7 in the green module; **D** MAP3K5 in the turquoise module; **E** GPBP1 in the yellow module

WGCNA analysis was conducted to further explore the co-expression network of DEGs and explain the significant transcriptome profile alterations. Firstly, a soft-thresholding power equaled 10 was set using the pickSoftThreshold program. Shown in the module assignment dendrogram plot (Fig. 3a), a total of five modules were identified by using the package *dynamicTreeCut* functioned to sort out a set of genes with high topological overlap index. Afterwards, the clinical phenotypes and module relationships were combined and the module trait associations were quantified. The results suggested that the brown and turquoise modules were correlated with the phenotypic comparison negatively, while the green, blue and yellow modules were associated with the phenotypic comparison positively (Fig. 3b). Next, hub gene of each module was identified by means of the package *TopHubInEachModule*. The hub genes of the blue, brown, green, turquoise and yellow modules in OLP tissues were RYK (receptor like tyrosine kinase), SLC8A1 (solute carrier family 8 member A1), WDR7 (WD repeat domain 7), MAP3K5 (mitogen-activated protein kinase kinase kinase 5) and GPBP1 (GC-rich promoter binding protein 1), respectively. To better visualize the intramodular connectivity of the hub genes in each module, we displayed the network applying the adjacency matrix of the eigengenes with the hub genes highlighted in each module (Fig. 4). Finally, the data processed by PEEPs algorithm which presents the inherent heterogeneity of gene transcription patterns in each individual specimen was shown in Table S3.

### Validation of mRNA expression of the top DEGs and hub genes in OLP

**Fig. 5:**
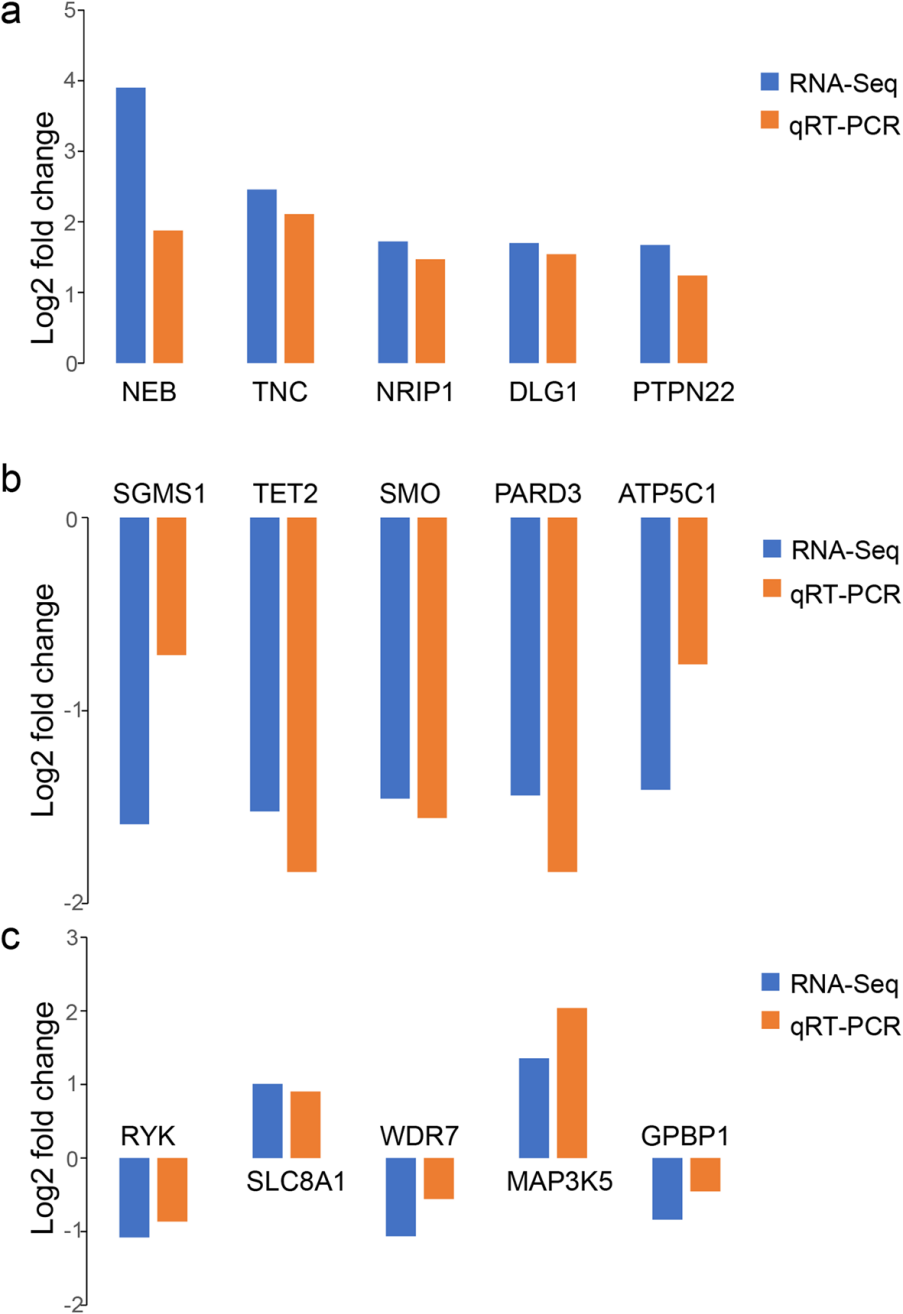
Comparisons between results of RNA-Seq and qRT-PCR verification for the top dysregulated DEGs and hub genes. A total of 15 genes were chosen for validation in another 10 pairs of OLP and normal oral mucosa. The relative mRNA expression levels were in keeping with the RNA-Seq. **a** Comparisons between results of RNA-Seq and qRT-PCR verification for the top 5 upregulated DEGs. **b** Comparisons between results of RNA-Seq and qRT-PCR verification for the top 5 downregulated DEGs. **c** Comparisons between results of RNA-Seq and qRT-PCR verification for the 5 hub genes

The mRNA expression of the five hub genes (RYK, SLC8A1, WDR7, MAP3K5, GPBP1) obtained from WGCNA analysis as well as the top DEGs (NEB, nebulin; TNC, tenascin c; NRIP1, nuclear receptor interacting protein 1; DLG1, discs large MAGUK scaffold protein 1; PTPN22, protein tyrosine phosphatase non-receptor type 22; SGMS1, sphingomyelin synthase 1; TET2, tet methylcytosine dioxygenase 2; SMO, smoothened, frizzled class receptor; PARD3, par-3 family cell polarity regulator; ATP5C1, ATP synthase f1 subunit gamma) from RNA-Seq results was detected by real-time PCR in comparisons between another 10 reticular OLP tissues and 10 normal oral mucosal tissues. The expression of NEB, TNC, NRIP1, DLG1, PTPN22 was notably increased in OLP compared to normal normal oral mucosa (Fig. 5a) while that of SGMS1, TET2, SMO, PARD3, ATP5C1 was decreased in OLP (Fig. 5b), which was consistent with the RNA-Seq data. As displayed in Fig. 5c the expression of SLC8A1 and MAP3K5 was higher in OLP than in the normal control; and the other 3 hub genes, RYK, WDR7 and GPBP1 were downregulated in OLP, although not all the them were statistically insignificant (SLC8A1: 1.87-fold increase, *p* = 0.0025; MAP3K5: 4.11-fold increase, *p* = 0.0001; RYK: 0.55-fold decrease, *p* = 0.0422; WDR7: 0.68-fold decrease, *p* = 0.1324; GPBP1: 0.73-fold decrease, *p* = 0.3399).

### Verification of CD3^+^ T cells in OLP by immunohistochemistry

**Fig. 6:**
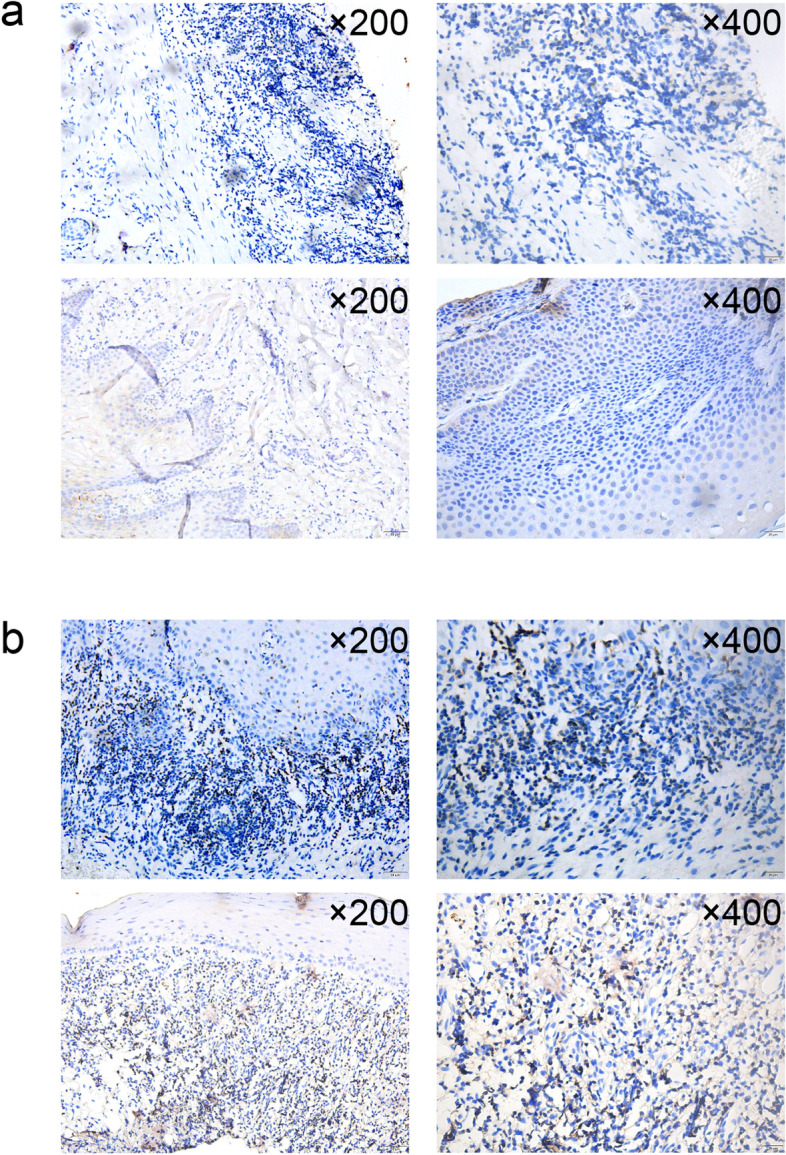
The expression of CD3^+^ T cells in OLP and normal tissues by immunohistochemistry. **A** The expression of CD3^+^ T cells in normal tissues; **B** The expression of CD3^+^ T cells in OLP

We examined the expression of CD3^+^ T cells in OLP and normal tissues by immunohistochemistry. The results in Fig. 6 showed a strong infiltrate of CD3^+^ T cells in OLP tissues. CD3^+^ T cells were obviously positively expressed in OLP compared to normal tissues and mainly gathered in the lamina propria, distributed around the epithelial basement membrane.

## Discussion

Oral lichen planus is a chronic disorder of oral mucosa with a global prevalence of 1.01% [10], and reticular OLP is the type with the highest prevalence. OLP is now generally recognized as a T-cell dysfunction-induced autoimmune disease, while the exact mechanism of this disease still remains poorly understood. To help further explain the underlying molecular mechanism in OLP, we integrated the RNA-Seq and bioinformatics to explore the transcriptional profiling of this disease.

A total of 153 DEGs were detected by RNA-Seq in this current study, of which 72 were increased while 81 were decreased in OLP tissues. The top 5 upregulated and downregulated genes in OLP were NEB, TNC, NRIP1, DLG1, PTPN22 and SGMS1, TET2, SMO, PARD3, ATP5C1, respectively. Different mRNA isoforms are deemed to take part in the proper regulation of diverse cells and the splicing defects lead to several known disorders. By means of *rSeqDiff* package, we detected the certain isoform variants for the top significant genes which were associated with the pathogenesis of OLP. These isoform transcripts may provide important clues for the alternative splice epigenetic mechanisms for the oral mucosa disease. Five co-expression modules were constructed by WGCNA method and hub genes of each module were identified as followed: RYK, SLC8A1, WDR7, MAP3K5, GPBP1. According to the GO and KEGG analysis, genes with aberrant expression in OLP were mainly associated with T cell receptor signaling pathway and the Wnt signaling pathway. Also, the IHC experiment have suggested the protein level related T cell activities significantly increased. Here, we considered that the RNA-sequence data may be originated from the T-cell enriched parts rather than the entire OLP tissues. These above-mentioned results were in line with the existing OLP pathogenesis, and provided new insights into the potential mechanism of OLP.

In OLP, a key pathogenesis is the activation of CD4^+^ T-helper and cytotoxic CD8^+^ lymphocytes along with the cytokines, resulting in keratinocytes apoptosis and degeneration of the epithelial basal layer [11]. T cell in OLP tend to proliferate rapidly and differentiate into distinct Th cell types, including Th1, Th2, Treg, Th17, etc. Growing evidence showed that Th1/Th2 imbalance influenced by Treg subset as well as the CD4^+^ Th subset Th17 took great part in immunopathology of OLP [12]. Nuclear receptor interacting protein 1 (NRIP1), also known as RIP140, was significantly increased in OLP. NRIP1 interacts with transcription factors and nuclear receptors, and previous studies focused on its molecular mechanism in regulating the immune response. Zhu Jun Yi et al. suggested that the overexpression of NRIP1 in macrophages promoted M1-like polarization and expansion in inflammatory diseases [13]. NRIP1 was found to modulate the transcription and secretion of pro-inflammatory cytokines by activating NF-κB [14] and this mechanism could also be found in autoimmune disease like psoriasis [15]. Interestingly, the NF-κB p65 signaling pathway was also activated and played a key role in the inflammatory response in OLP [16]. DLG1, which was one of the top high expressed DEGs in OLP tissues, is a member of the family of molecular scaffolding proteins and encoded a multi-domain scaffolding protein required for normal development. Altered expression of DLG1 is observed in several immunological disorders. Dlg1 functions as a positive regulator of immunity and facilitated T cell receptor (TCR) signal transduction and T cell function. T cells lacking proper Dlgh1 expression were deficient in coupling antigenic stimulation resulting in incapability of producing cytokine [17]. TET2, which was the most significantly downregulated gene, is universally considered as a tumor-suppressor gene and could modulate Th1 and Th17 cell differentiation and T-cell cytokine production in vivo in autoimmune diseases [18]. Earlier study showed that mutation of TET2 caused aberrant CD4^+^ T cell proliferation and disturbance of T cell homeostasis [19], which was closely related to the occurence of OLP. Besides, Tet2 downregulation was vital for 5-hydroxymethylcytocine (5hmC) regulation in adult T-cell leukemia/lymphoma (ATLL) progression [20] and Tet2-KO mice developed myeloid cancers with an incidence rate of about 30% [21]. These results demonstrated that TET2 was strongly associated with immune responses. MAP3K5 (also named ASK1), which was the hub gene in turquoise module acquired from WGCNA, is an important member of mitogen-activated protein kinase (MAPK) family and plays crucial roles in physiological processes associated with autoimmune diseases. Recently, S.J. Mnich et al. found that as an essential component for the development of rheumatoid arthritis, MAP3K5 participated in the tumor necrosis factor-alpha (TNF-α)-induced production of inflammatory mediators [22] and MAP3K5 deficiency could reduce neuroinflammation in experimental autoimmune encephalomyelitis [23]. In addition, in aberrant ribosomal biogenesis, activated MAP3K5 pathways regulated the stability and activity of p53 protein [24], which acted as a transcription factor mediating the induction of cell apoptosis [25]. Taken into consideration the fact that the expression of p53 has been reported upregulated in OLP tissues [26, 27], the interaction between MAP3K5 and p53 may play roles in the pathogenesis of OLP.

Wnt signaling played key roles during cell proliferation and differentiation and was implicated in multiple autoimmune diseases due to its participation in the development of T cells. In OLP, Wnt3 has been featured with positive expression in cytoplasm and lack of nuclear staining, suggesting a potential involvement of Wnt signaling pathway in OLP progression [28]. RYK, the hub gene in blue module, belonged to the atypical receptor tyrosine kinase family and was a receptor of Wnt ligand Wnt5a. The interaction of Wnt ligands and RYK originated the downstream signaling cascades critical for immune regulation [29]. Besides, Tenascin C (TNC), ranking as the 2nd top upregulated gene in OLP, encoded an extracellular matrix protein (ECM) and was a promoter of tumor metastasis with multiple functions. TNC expression positively linked to cancer progression and cell proliferation. TNC downregulated Dick-kopf-1 (DKK1), a Wnt inhibitor by blocking of actin stress fiber formation, thus activating Wnt signaling and induced Wnt target genes in tumor and endothelial cells [30]. Nevertheless, the high expression of TNC induced by Wnt/beta-catenin activation, acting as a downstream target, leaded to a more tumorigenic cell state in Ewing sarcoma [31]. The above researches demonstrated that the interaction between TNC and Wnt signaling participated in the modulation of tumor progression and invasion. Nevertheless, to the best of our knowledge, the rest of the key genes have not been reported highly involved in T cell regulation and inflammation or Wnt signaling pathway.

In 2017, Junjun Chen et al. [32] identified 94 differentially expressed miRNAs (DEMs) and 599 DEGs in total in OLP tissues with RNA sequencing and constructed a miRNA–mRNA network regulating pathogenetic process. Using GO and KEGG pathway analysis, the DEGs were significantly enriched in immune-related functional ontology and pathways. The study focused mainly on the potential mechanisms that may contribute to the DEMs in inflammatory events. In current study, we illustrated the immune mediating into the pathogenesis of OLP from the perspective of gene co-expression pattern using group-wise and WGCNA analysis, which was distinct from the previous study.

There were limitations in our study. Firstly, the samples size was small. Only reticular OLP tissues were collected and there was a lack of OLP tissues of erosive and erythematous types. Secondly, we did not validate the functions of the key genes as well as the potential signaling pathways in vitro or in vivo.

All in all, in this study, we strictly selected the matched samples of OLP and normal oral mucosal tissues and a range of genes were observed differentially expressed in OLP tissues. Subsequently, by application of the bioinformatic analysis method, our research also confirmed the key roles of biological activities associated with T cell regulation and inflammation and Wnt signaling pathway that have been reported in earlier studies on the molecular mechanisms of OLP and provided a theoretical basis for the study of novel drug targets.

## Conclusions

Taken together, we constructed a transcriptional profiling of OLP using RNA-Seq and 153 differentially expressed genes were detected. Here, with the combination of the results of GO enrichment annotation analysis, KEGG pathway and WGCNA analysis based on the DEGs, biological process including T cell regulation and inflammation and *Wnt* signaling pathway were identified highly correlated to the pathogenesis of OLP. Further researches are required to explore the molecular mechanism of the key genes as the candidate biomarkers in this disease.

## Supplementary Information

**Additional file 1: Table S1.** The most significant dysregulated genes identified from transcriptional profiling. **Table S2.** The GO terms including BP, CC and MP from the dysregulated genes. **Table S3.** Genes perturbed in each individual specimen by PEEPs algorithm. **Table S4.** Primer used in qRT-PCR.

## Data Availability

The raw data of RNA sequence is available from the corresponding author on reasonable request.

## Abbreviations

BP: Biological process
CC: Cell component
DEGs: Differentially expressed genes
GO: Gene Ontology
MF: Molecular function
OLP: Oral lichen planus
OPMD: Oral potential malignant disorder
PEEPs: Personalized expression perturbation profiles
WGCNA: Weighted gene co-expression network analysis
