# Supplementary material for: RNA-Seq based transcriptome analysis in oral lichen planus
Source: Hereditas. 2021 Oct 6;158:39. doi: 10.1186/s41065-021-00202-z (PMC8495917; doi:10.1186/s41065-021-00202-z)
Supplement: Supplementary file 1 — Additional file 1: Table S1. The most significant dysregulated genes identified from transcriptional profiling. Table S2. The GO terms including BP, CC and MP from the dysregulated genes. Table S3. Genes perturbed in each individual specimen by PEEPs algorithm. Table S4. Primer used in qRT-PCR. [file 41065_2021_202_MOESM1_ESM.docx]

**Table S1 The most significant dysregulated genes identified from transcriptional profiling.**

| Gene | Description | Gene ID | Regulation | Fold change | p-value | Isoform name | Isoform difference |
| --- | --- | --- | --- | --- | --- | --- | --- |
| NEB | nebulin | 4703 | Up | 14.8994992 | 0.017850442 | uc002txq.3  uc002txr.3  uc002txu.3  uc010zbz.2  uc021vrb.1 | 0.288521759  0.297976366  0.132899109  0.363031129  0.138743315 |
| TNC | tenascin C | 3371 | Up | 5.486877235 | 0.037742412 | uc004bjj.4  uc010mvf.3 | 0.181608734  0.066240498 |
| NRIP1 | nuclear receptor interacting protein 1 | 8204 | Up | 3.298052886 | 0.041012046 | uc002yjx.2 | 0.343269299 |
| DLG1 | discs large MAGUK scaffold protein 1 | 1739 | Up | 3.242861896 | 0.038952227 | uc011bub.2 | 0.032116994 |
| PTPN22 | protein tyrosine phosphatase non-receptor type 22 | 26191 | Up | 3.186197827 | 0.016261397 | uc001eds.3 | 0.414548429 |
| SGMS1 | sphingomyelin synthase 1 | 259230 | Down | 0.332532325 | 0.010487991 | uc001jje.3 | 0.449811497 |
| TET2 | tet methylcytosine dioxygenase 2 | 54790 | Down | 0.348102841 | 0.014933145 | uc010ilp.2  uc003hxk.3 | 0.187150816  0.5 |
| SMO | smoothened, frizzled class receptor | 6608 | Down | 0.364485425 | 0.002777887 | uc003vor.3 | 0.149884197 |
| PARD3 | par-3 family cell polarity regulator | 56288 | Down | 0.3688966 | 0.009925126 | uc010qej.2  uc010qek.2 | 0.211913879  0.333799439 |
| ATP5C1 | ATP synthase F1 subunit gamma | 509 | Down | 0.376254014 | 0.01481178 | uc001iju.3 | 0.451156523 |

**Table S2 The GO terms including BP, CC and MP from the dysregulated genes**

| Function | Rank | GO.ID | Description | BgRatio | pvalue | p.adjust | geneID | Count |
| --- | --- | --- | --- | --- | --- | --- | --- | --- |
| MF | 1 | GO:0003714 | transcription corepressor activity | 238/176c96 | 0.000230269 | 0.090495577 | NRIP1/SFMBT2/N4BP2L2/CTBP1/ZMYND11/CBFA2T2/ATF7IP/GON4L/RBBP8 | 9 |
|  | 2 | GO:0030507 | spectrin binding | 28/17696 | 0.001771619 | 0.206760041 | CAMSAP1/ANK3/MYO10 | 3 |
|  | 3 | GO:0140142 | nucleocytoplasmic carrier activity | 31/17696 | 0.002385228 | 0.206760041 | KPNA5/NUP42/TNPO3 | 3 |
|  | 4 | GO:0016791 | phosphatase activity | 270/17696 | 0.002443236 | 0.206760041 | DLG1/PTPN22/CTDP1/PIKFYVE/PPP3CC/UBASH3A/PTPN13/PPP4R1 | 8 |
|  | 5 | GO:0015631 | tubulin binding | 336/17696 | 0.002630535 | 0.206760041 | HTT/CCDC66/CLASP2/FCHO2/FYN/TBCEL/SBDS/CAMSAP1/MTCL1 | 9 |
|  | 6 | GO:0044325 | ion channel binding | 124/17696 | 0.004468033 | 0.214437218 | DLG1/SLC8A1/HTT/FYN/ANK3 | 5 |
|  | 7 | GO:0005547 | phosphatidylinositol-3,4,5-trisphosphate binding | 39/17696 | 0.004611174 | 0.214437218 | ARAP2/MYO10/PARD3 | 3 |
|  | 8 | GO:0005089 | Rho guanyl-nucleotide exchange factor activity | 79/17696 | 0.004885282 | 0.214437218 | ARHGEF4/ARHGEF12/ARHGEF28/DOCK1 | 4 |
|  | 9 | GO:0004721 | phosphoprotein phosphatase activity | 182/17696 | 0.005060833 | 0.214437218 | DLG1/PTPN22/CTDP1/PPP3CC/PTPN13/PPP4R1 | 6 |
|  | 10 | GO:0050681 | androgen receptor binding | 44/17696 | 0.006475735 | 0.214437218 | NRIP1/FOXP1/KDM1A | 3 |
|  | 11 | GO:0005088 | Ras guanyl-nucleotide exchange factor activity | 137/17696 | 0.006782278 | 0.214437218 | ARHGEF4/RAB3IP/ARHGEF12/ARHGEF28/DOCK1 | 5 |
|  | 12 | GO:0042393 | histone binding | 197/17696 | 0.007376641 | 0.214437218 | SFMBT2/DNAJC2/SMARCA5/SMARCC1/KDM1B/ZMYND11 | 6 |
|  | 13 | GO:0051015 | actin filament binding | 198/17696 | 0.007554265 | 0.214437218 | NEB/TNNC1/SLC6A4/UTRN/SYNE2/MYO10 | 6 |
|  | 14 | GO:0035254 | glutamate receptor binding | 47/17696 | 0.007780628 | 0.214437218 | DLG1/FYN/CANX | 3 |
|  | 15 | GO:0035258 | steroid hormone receptor binding | 92/17696 | 0.008335839 | 0.214437218 | NRIP1/FOXP1/CNOT1/KDM1A | 4 |
|  | 16 | GO:0034185 | apolipoprotein binding | 17/17696 | 0.00927591 | 0.214437218 | LRP6/CANX | 2 |
|  | 17 | GO:0042813 | Wnt-activated receptor activity | 17/17696 | 0.00927591 | 0.214437218 | LRP6/RYK | 2 |
| CC | 1 | GO:0031253 | cell projection membrane | 326/19717 | 0.000291899 | 0.058358164 | DLG1/CLASP2/ARHGEF4/UTRN/CD44/SLC11A2/SYNE2/DHRS3/MYO10/SMO | 10 |
|  | 2 | GO:0031527 | filopodium membrane | 18/19717 | 0.000370174 | 0.058358164 | UTRN/SYNE2/MYO10 | 3 |
|  | 3 | GO:0016234 | inclusion body | 82/19717 | 0.000500213 | 0.058358164 | SFMBT2/PICALM/HTT/ATXN1/SYNE2 | 5 |
|  | 4 | GO:0005938 | cell cortex | 308/19717 | 0.000828718 | 0.072512839 | SPINK5/MYO9B/CLASP2/ASPH/UTRN/CTBP1/EXOC6B/MYO10/PARD3 | 9 |
|  | 5 | GO:0000407 | phagophore assembly site | 32/19717 | 0.002073706 | 0.145159438 | ATG2B/ILRUN/PIK3C3 | 3 |
|  | 6 | GO:0030027 | lamellipodium | 193/19717 | 0.004538458 | 0.20997163 | KLHL2/RAB3IP/CD44/SYNE2/PTPN13/MYO10 | 6 |
|  | 7 | GO:0031252 | cell leading edge | 403/19717 | 0.005074705 | 0.20997163 | CLASP2/ARHGEF4/KLHL2/RAB3IP/SLK/CD44/SYNE2/PTPN13/MYO10 | 9 |
|  | 8 | GO:0099523 | presynaptic cytosol | 14/19717 | 0.005384096 | 0.20997163 | HTT/PPP3CC | 2 |
|  | 9 | GO:0030122 | AP-2 adaptor complex | 15/19717 | 0.006180101 | 0.20997163 | PICALM/FCHO2 | 2 |
|  | 10 | GO:0033268 | node of Ranvier | 15/19717 | 0.006180101 | 0.20997163 | DLG1/ANK3 | 2 |
|  | 11 | GO:0030128 | clathrin coat of endocytic vesicle | 16/19717 | 0.007026254 | 0.20997163 | PICALM/FCHO2 | 2 |
|  | 12 | GO:0014704 | intercalated disc | 50/19717 | 0.007382694 | 0.20997163 | DLG1/SLC8A1/ANK3 | 3 |
|  | 13 | GO:0045178 | basal part of cell | 51/19717 | 0.007798946 | 0.20997163 | CLASP2/SLC11A2/ANK3 | 3 |
|  | 14 | GO:0030175 | filopodium | 104/19717 | 0.009612528 | 0.213726104 | DEF6/UTRN/SYNE2/MYO10 | 4 |
|  | 15 | GO:0031965 | nuclear membrane | 296/19717 | 0.009665884 | 0.213726104 | DNAJC2/NUP42/MYOF/NUP205/SYNE2/PUM2/SMOX | 7 |
|  | 16 | GO:0016328 | lateral plasma membrane | 57/19717 | 0.010585183 | 0.213726104 | DLG1/ANK3/MTCL1 | 3 |
|  | 17 | GO:0030132 | clathrin coat of coated pit | 20/19717 | 0.010895721 | 0.213726104 | PICALM/FCHO2 | 2 |
|  | 18 | GO:0044232 | organelle membrane contact site | 21/19717 | 0.011980233 | 0.213726104 | ESYT2/CANX | 2 |
|  | 19 | GO:0031258 | lamellipodium membrane | 22/19717 | 0.013110008 | 0.213726104 | CD44/SYNE2 | 2 |
|  | 20 | GO:0045121 | membrane raft | 315/19717 | 0.013260046 | 0.213726104 | DLG1/SLC6A4/PIKFYVE/LRP6/FYN/MYOF/SMO | 7 |
|  | 21 | GO:0098857 | membrane microdomain | 316/19717 | 0.013472555 | 0.213726104 | DLG1/SLC6A4/PIKFYVE/LRP6/FYN/MYOF/SMO | 7 |
|  | 22 | GO:0045211 | postsynaptic membrane | 323/19717 | 0.015029198 | 0.213726104 | DLG1/PICALM/SLC8A1/SLC6A4/UTRN/CANX/ANK3 | 7 |
|  | 23 | GO:0099522 | region of cytosol | 24/19717 | 0.01550224 | 0.213726104 | HTT/PPP3CC | 2 |
|  | 24 | GO:0005925 | focal adhesion | 405/19717 | 0.016014875 | 0.213726104 | TNC/SLC6A4/USP33/CLASP2/HSPG2/PPFIA1/CD44/SYNE2 | 8 |
|  | 25 | GO:0098589 | membrane region | 328/19717 | 0.016216979 | 0.213726104 | DLG1/SLC6A4/PIKFYVE/LRP6/FYN/MYOF/SMO | 7 |
|  | 26 | GO:0098793 | presynapse | 491/19717 | 0.016997489 | 0.213726104 | PICALM/HTT/SLC6A4/PPP3CC/FCHO2/CANX/CTBP1/PPFIA1/WDR7 | 9 |
|  | 27 | GO:0044304 | main axon | 68/19717 | 0.017019363 | 0.213726104 | DLG1/ANK3/PARD3 | 3 |
|  | 28 | GO:0030055 | cell-substrate junction | 412/19717 | 0.017563649 | 0.213726104 | TNC/SLC6A4/USP33/CLASP2/HSPG2/PPFIA1/CD44/SYNE2 | 8 |
|  | 29 | GO:0016529 | sarcoplasmic reticulum | 71/19717 | 0.019078916 | 0.213726104 | ASPH/SYNE2/ANK3 | 3 |
|  | 30 | GO:0044291 | cell-cell contact zone | 71/19717 | 0.019078916 | 0.213726104 | DLG1/SLC8A1/ANK3 | 3 |
|  | 31 | GO:0048786 | presynaptic active zone | 71/19717 | 0.019078916 | 0.213726104 | CANX/CTBP1/PPFIA1 | 3 |
|  | 32 | GO:0030018 | Z disc | 132/19717 | 0.021319042 | 0.213726104 | NEB/SLC8A1/SYNE2/ANK3 | 4 |
|  | 33 | GO:0030125 | clathrin vesicle coat | 29/19717 | 0.02222148 | 0.213726104 | PICALM/FCHO2 | 2 |
|  | 34 | GO:0030131 | clathrin adaptor complex | 29/19717 | 0.02222148 | 0.213726104 | PICALM/FCHO2 | 2 |
|  | 35 | GO:0097060 | synaptic membrane | 432/19717 | 0.022580355 | 0.213726104 | DLG1/PICALM/SLC8A1/SLC6A4/UTRN/FCHO2/CANX/ANK3 | 8 |
|  | 36 | GO:0031594 | neuromuscular junction | 76/19717 | 0.022804954 | 0.213726104 | DLG1/UTRN/ANK3 | 3 |
|  | 37 | GO:0042383 | sarcolemma | 136/19717 | 0.02348242 | 0.213726104 | DLG1/SLC8A1/UTRN/ANK3 | 4 |
|  | 38 | GO:0017053 | transcription repressor complex | 77/19717 | 0.023594246 | 0.213726104 | N4BP2L2/CTBP1/RBBP8 | 3 |
|  | 39 | GO:0030017 | sarcomere | 204/19717 | 0.023815194 | 0.213726104 | NEB/TNNC1/SLC8A1/SYNE2/ANK3 | 5 |
|  | 40 | GO:0098858 | actin-based cell projection | 208/19717 | 0.02562256 | 0.217085477 | DEF6/UTRN/CD44/SYNE2/MYO10 | 5 |
|  | 41 | GO:0005901 | caveola | 80/19717 | 0.026050257 | 0.217085477 | LRP6/MYOF/SMO | 3 |
|  | 42 | GO:0016528 | sarcoplasm | 80/19717 | 0.026050257 | 0.217085477 | ASPH/SYNE2/ANK3 | 3 |
|  | 43 | GO:0031674 | I band | 143/19717 | 0.027579188 | 0.224481764 | NEB/SLC8A1/SYNE2/ANK3 | 4 |
| BP | 1 | GO:0043392 | negative regulation of DNA binding | 54/18670 | 7.44E-05 | 0.112187482 | ILRUN/IFI16/FBXW7/KDM1A/SMO | 5 |
|  | 2 | GO:0071679 | commissural neuron axon guidance | 12/18670 | 0.000108152 | 0.112187482 | NFIB/RYK/SMO | 3 |
|  | 3 | GO:1905475 | regulation of protein localization to membrane | 187/18670 | 0.00014306 | 0.112187482 | DLG1/PICALM/PPP3CC/FYN/PPFIA1/MAPK8/ANK3/MTCL1 | 8 |
|  | 4 | GO:0006914 | autophagy | 496/18670 | 0.000200621 | 0.112187482 | PTPN22/HTT/ATG2B/USP33/ILRUN/PLEKHM1/PIKFYVE/IFI16/STAM/FBXW7/PIK3C3/MAPK8/MTCL1 | 13 |
|  | 5 | GO:0061919 | process utilizing autophagic mechanism | 496/18670 | 0.000200621 | 0.112187482 | PTPN22/HTT/ATG2B/USP33/ILRUN/PLEKHM1/PIKFYVE/IFI16/STAM/FBXW7/PIK3C3/MAPK8/MTCL1 | 13 |
|  | 6 | GO:0031098 | stress-activated protein kinase signaling cascade | 315/18670 | 0.000249454 | 0.116245573 | DLG1/PTPN22/MAP3K5/ERCC6/RELL1/SLK/NEK4/ZMYND11/MAPK8/KLHDC10 | 10 |
|  | 7 | GO:0022612 | gland morphogenesis | 120/18670 | 0.000433896 | 0.135137256 | TNC/AREG/LRP6/FBXW7/FGFR1/NFIB | 6 |
|  | 8 | GO:0051100 | negative regulation of binding | 169/18670 | 0.000455176 | 0.135137256 | USP33/ILRUN/IFI16/FBXW7/MAPK8/KDM1A/SMO | 7 |
|  | 9 | GO:1905477 | positive regulation of protein localization to membrane | 122/18670 | 0.000473851 | 0.135137256 | DLG1/PPP3CC/FYN/MAPK8/ANK3/MTCL1 | 6 |
|  | 10 | GO:0051101 | regulation of DNA binding | 124/18670 | 0.000516617 | 0.135137256 | ILRUN/IFI16/FBXW7/MAPK8/KDM1A/SMO | 6 |
|  | 11 | GO:1900744 | regulation of p38MAPK cascade | 47/18670 | 0.000559657 | 0.135137256 | DLG1/PTPN22/MAP3K5/RELL1 | 4 |
|  | 12 | GO:0090316 | positive regulation of intracellular protein transport | 176/18670 | 0.000579988 | 0.135137256 | PPP3CC/FYN/FBXW7/MAPK8/ANK3/MTCL1/SMO | 7 |
|  | 13 | GO:0038066 | p38MAPK cascade | 53/18670 | 0.00088496 | 0.179492898 | DLG1/PTPN22/MAP3K5/RELL1 | 4 |
|  | 14 | GO:0021537 | telencephalon development | 249/18670 | 0.000965799 | 0.179492898 | BCL11B/SLC8A1/LRP6/SYNE2/NFIB/RYK/KDM1A/SMO | 8 |
|  | 15 | GO:0048488 | synaptic vesicle endocytosis | 56/18670 | 0.001089394 | 0.179492898 | PICALM/PPP3CC/FCHO2/CANX | 4 |
|  | 16 | GO:0140238 | presynaptic endocytosis | 56/18670 | 0.001089394 | 0.179492898 | PICALM/PPP3CC/FCHO2/CANX | 4 |
|  | 17 | GO:0030900 | forebrain development | 381/18670 | 0.001091337 | 0.179492898 | BCL11B/SLC8A1/LRP6/FYN/FGFR1/SYNE2/NFIB/RYK/KDM1A/SMO | 10 |
|  | 18 | GO:0002831 | regulation of response to biotic stimulus | 400/18670 | 0.001568268 | 0.218082862 | PTPN22/PSMB1/FOXP1/SPINK5/ILRUN/ERCC6/IFI16/FYN/PUM2/YTHDF2 | 10 |
|  | 19 | GO:0050855 | regulation of B cell receptor signaling pathway | 29/18670 | 0.001624128 | 0.218082862 | PTPN22/FOXP1/ELF2 | 3 |
|  | 20 | GO:0051642 | centrosome localization | 29/18670 | 0.001624128 | 0.218082862 | DLG1/SYNE2/PARD3 | 3 |
|  | 21 | GO:2001233 | regulation of apoptotic signaling pathway | 406/18670 | 0.001749944 | 0.218082862 | HTT/PPP3CC/FYN/FBXW7/FGFR1/ZMYND11/MAPK8/CD44/KDM1A/SGMS1 | 10 |
|  | 22 | GO:0061842 | microtubule organizing center localization | 30/18670 | 0.001793958 | 0.218082862 | DLG1/SYNE2/PARD3 | 3 |
|  | 23 | GO:0090314 | positive regulation of protein targeting to membrane | 30/18670 | 0.001793958 | 0.218082862 | FYN/ANK3/MTCL1 | 3 |

**Table S3 Genes perturbed in each individual specimen by PEEPs algorithm**

| Case number | Genes |
| --- | --- |
| R17004387LR01 | "TNC" "DLG1" "PTPN22" "BCL11B" "KPNA5" "MAP3K5" "PSMB1" "SFMBT2" "FOXP1" "PICALM" "CTDP1" "ABHD2" "UGP2" "ESYT2" "DNAJC2" "MYO9B" "ARAP2" "LZIC" "SLC8A1" "HTT" "KLHL8" "NUPL2" "ATG2B" "USP33"  "N4BP2L2" "BRWD1" "C6orf106" "PLEKHM1" "THSD1" "RBM33" "DEF6" "KLHL2" "KANSL1L" "PPP3CC" "RAB3IP" "FCHO2"  "SMARCA5" "IFI16" "SLK" "ARHGAP5" "TNPO3" "PPFIA1" "ZBTB44" "EXOC6B" "KLHDC10" "ATF7IP" "HPS5" "CLIP4"  "DCUN1D4" "TRPS1" "SMO" |
| R17004388LR01 | "NEB" "TNC" "NRIP1" "PTPN22" "KPNA5" "SFMBT2" "ZRANB1" "FOXP1" "ESYT2" "MYO9B" "ARAP2" "SLC8A1"  "HTT" "KLC1" "KLHL8" "NUPL2" "FGGY" "ATXN10" "STAU2" "ATG2B" "CLK1" "BRWD1" "CCDC9" "CCDC66"  "C6orf106" "ERCC6" "THSD1" "RBM33" "UTRN" "OGDH" "NDUFA10" "FAM73A" "SMARCC1" "RSL1D1" "HPS5" "RYK" "PTPN13" "TRPS1" "SMO" |
| R17004389LR01 | "NEB" "TNC" "NRIP1" "DLG1" "PTPN22" "BCL11B" "KPNA5" "MAP3K5" "PSMB1" "SFMBT2" "ZRANB1" "FOXP1" "PROSC" "PICALM" "CTDP1" "UGP2" "ESYT2" "AREG" "DNAJC2" "MYO9B" "ARAP2" "LZIC" "SLC8A1" "CCDC134" "HTT" "KLC1" "NUPL2" "FGGY" "ATG2B" "USP33" "N4BP2L2" "BRWD1" "SDF4" "ERCC6" "ARHGEF4" "RBM33" "DEF6" "UTRN" "RELL1" "SNRK" "STAM" "TNPO3" "ELF2" "RSL1D1" "EXOC6B" "SLC11A2" "DCUN1D4" "NDUFS1" "RYK" "SOGA2" "PPP4R1" "PARD3" |
| TI1707200244LR01 | "NEB" "TNC" "NRIP1" "DLG1" "PTPN22" "BCL11B" "KPNA5" "MAP3K5" "PSMB1" "SFMBT2" "FOXP1" "PROSC"  "PICALM" "CTDP1" "ABHD2" "UGP2" "ESYT2" "DNAJC2" "LZIC" "SLC8A1" "CCDC134" "KLC1" "KLHL8" "NUPL2"  "FGGY" "ATXN10" "STAU2" "ATG2B" "USP33" "CLK1" "N4BP2L2" "KIAA0146" "SDF4" "CLASP2" "C6orf106" "ERCC6"  "PIKFYVE" "THSD1" "RELL1" "PPP3CC" "FCHO2" "SMARCA5" "PCMTD1" "CANX" "ARHGAP5" "CTBP1" "PTGR1" "FNDC3B"  "PPFIA1" "PDS5A" "ATF7IP" "SLC11A2" "DCUN1D4" "PTPN13" "YTHDF2" "SOGA2" "ATP5C1" "SMO" |
| TI1707200245LR01 | "NEB" "NRIP1" "MAP3K5" "ZRANB1" "PROSC" "SPINK5" "ESYT2" "DNAJC2" "LZIC" "KLHL8" "ATXN10" "STAU2" "N4BP2L2" "KIAA0146" "C6orf106" "PLEKHM1" "PIKFYVE" "RBM33" "ASPH" "KLHL2" "KANSL1L" "FAM73A" "SMARCC1" "PPFIA1" "MAPK8" "ATF7IP" "ITM2B" "HPS5" "DCUN1D4" "NDUFS1" "PTPN13" "TRPS1" "SOGA2" |
| TI1707200246LR01 | "NEB" "DLG1" "BCL11B" "MAP3K5" "PSMB1" "ZRANB1" "PROSC" "PICALM" "SPINK5" "CTDP1" "ABHD2" "UGP2" "ESYT2" "DNAJC2" "ARAP2" "LZIC" "CCDC134" "HTT" "KLC1" "ATXN10" "ATG2B" "USP33" "CLK1" "N4BP2L2" "KIAA0146" "CCDC66" "C6orf106" "PLEKHM1" "ARHGEF4" "RBM33" "ASPH" "LRP6" "PPP3CC" "EPRS" "FCHO2" "SMARCC1" "PCMTD1" "LIFR" "RSL1D1" "PPFIA1" "KLHDC10" "ATF7IP" "SLC11A2" "HPS5" "NDUFS1" "RYK" "SOGA2" "SMO" |
| TI1707200247LR01 | "PIKFYVE" |
| TI1707200252LR01 | 0 |
| R17004390LR01 | 0 |
| R17004391LR01 | "SBDS" |
| R17004392LR01 | "KLC1" |
| R17004393LR01 | 0 |

**Table S4 Primer used in qRT-PCR**

| Gene | Forward（5’-3’） | Reverse（5’-3’） |
| --- | --- | --- |
| *NEB* | GGACACCCGTTACATGAGCA | CCGATACAGTCTCTCGCTGG |
| *TNC* | TGGCATCGGAGAATGCCTTT | TCCGGTTCGGCTTCTGTAAC |
| *NRIP1* | ACACAGCCAGAAGATGCACA | TGTTCGTCTGTCTCCAAGCTC |
| *DLG1* | CTGCTGAGTGAGGTTGAGGG | GGAAGAGGGCACACACCTTG |
| *PTPN22* | TTCTTCTCCCCCACCTCCTC | TTCTGCAGGCTTGTTTGGTG |
| *SGMS1* | GAAAGCGTTCGCACCAGC | GCTGTCGTCACGTTGCACT |
| *TET2* | AGCAAGATGGCTGCCCTTTA | ATGTTTGCCAGCCTCGTTCT |
| *SMO* | GCGCGAGGAGGAGCG | CATTCCGGAGGCCCGAC |
| *PARD3* | TACCAGCTGTCCCCTACAGT | TGGACTCAAAGAGCAGTCGG |
| *RYK* | CAACCACTTCTACGCGTGTG | AGGATAACCTAAGGAGCTGGTG |
| *SLC8A1* | TGAATGAGCTTGGTGGCTTC | CTCTTTGCTGGTCAGTGGCT |
| *WDR7* | AAAGTAGGCGCCGTTACCAA | GGCTGTTTCCTGCCATTGTG |
| *MAP3K5* | CCCTGAGATCCCAGAGTCCA | TGATCCAGCTGAAAGAGCTGA |
| *GPBP1* | AGAACTTGGCTAATTCGGGAGA | CCTCATGGCAAGTCCAAATTCC |
| *ATP5C1* | GCTGTGGCTACCATGTTCTC | TTCGAACTTGAATCCATTGCGG |
